# G-Protein Coupled Receptor 30 (GPR30): A Novel Regulator of Endothelial Inflammation

**DOI:** 10.1371/journal.pone.0052357

**Published:** 2012-12-20

**Authors:** Subhadeep Chakrabarti, Sandra T. Davidge

**Affiliations:** 1 Department of Obstetrics & Gynecology, University of Alberta, Edmonton, Alberta, Canada; 2 Women and Children’s Health Research Institute, University of Alberta, Edmonton, Alberta, Canada; 3 Cardiovascular Research Centre, University of Alberta, Edmonton, Alberta, Canada; 4 Mazankowski Alberta Heart Institute, Edmonton, Alberta, Canada; 5 Department of Physiology, University of Alberta, Edmonton, Alberta, Canada; McMaster University, Canada

## Abstract

Estrogen, the female sex hormone, is known to exert anti-inflammatory and anti-atherogenic effects. Traditionally, estrogen effects were believed to be largely mediated through the classical estrogen receptors (ERs). However, there is increasing evidence that G-protein coupled receptor 30 (GPR30), a novel estrogen receptor, can mediate many estrogenic effects on the vasculature. Despite this, the localization and functional significance of GPR30 in the human vascular endothelium remains poorly understood. Given this background, we examined the subcellular location and potential anti-inflammatory roles of GPR30 using human umbilical vein endothelial cells as a model system. Inflammatory changes were induced by treatment with tumor necrosis factor (TNF), a pro-inflammatory cytokine involved in atherogenesis and many other inflammatory conditions. We found that GPR30 was located predominantly in the endothelial cell nuclei. Treatment with the selective GPR30 agonist G-1 partially attenuated the TNF induced upregulation of pro-inflammatory proteins such as intercellular cell adhesion molecule-1 (ICAM-1) and vascular cell adhesion molecule-1 (VCAM-1). This effect was completely abolished by the selective GPR30 antagonist G-15, suggesting that it was indeed mediated in a GPR30 dependent manner. Interestingly, estrogen alone had no effects on TNF-treated endothelium. Concomitant activation of the classical ERs blocked the anti-inflammatory effects of G-1, indicating opposing effects of GPR30 and the classical ERs. Our findings demonstrate that endothelial GPR30 is a novel regulator of the inflammatory response which could be a potential therapeutic target against atherosclerosis and other inflammatory diseases.

## Introduction

Premenopausal women are relatively protected against atherosclerosis and its complications such as myocardial infarction and stroke compared to age-matched men [1], [2]. Inflammatory changes in the vascular endothelium underlie the pathogenesis of atherosclerosis. Higher levels of the female sex hormone estrogen are believed to exert anti-inflammatory and anti-atherogenic effects on the female vasculature. However, clinical trials of exogenous estrogen therapy have yielded equivocal results, suggesting a more nuanced role for estrogen on the vascular system [3], [4]. 17-β-Estradiol (E2), the common form of estrogen in the body mediates its effects through at least 3 different receptors- the classical estrogen receptors (ERs), namely, ERα and ERβ as well as G-protein coupled receptor 30 (GPR30, also known as G-protein coupled estrogen receptor 1/GPER) [5]–[7]. Since these receptors differ in tissue distribution, subcellular locations and signaling roles, it is likely that relative differences in estrogen receptor expression and activity may affect the estrogenic effects on the vasculature.

Although GPR30 was identified as an orphan G-protein coupled receptor (GPCR) in 1997 [8], its role as a novel estrogen receptor was clearly established only in 2005 [9]. Since then the discovery of selective GPR30 modulators and the creation of several knockout mouse models has resulted in an explosive increase in data regarding the role of GPR30 on various systems under both physiological and pathological states [10]–[12]. Several groups have now demonstrated GPR30 expression in the vascular endothelium of rodents where it appears to mediate vasorelaxant functions [13], [14]. GPR30 activation has also been shown to mediate anti-inflammatory effects in rodent models of multiple sclerosis and ischemia-reperfusion injury [15]–[18]. These findings have suggested that GPR30 could be a potential target for new therapies against a range of inflammatory diseases including those affecting the vascular system.

However, the role of GPR30 in the human endothelium remains poorly understood. The GPR30 agonist G-1 has been shown to induce actin polymerization and reduce cellular proliferation in human umbilical vein endothelial cells (HUVECs) suggesting the presence of a functional GPR30 in these cells [19]. The GPR30 mRNA has also been demonstrated in human endothelium yet data on the existence of GPR30 protein remains sparse [20]. Indeed, only a single publication has shown the presence of GPR30 protein in human endothelium to date and the functional significance of this endothelial GPR30 in humans is largely unknown [21]. Given this background, we aimed to characterize the subcellular localization of GPR30 in human endothelial cells and examine its role in regulating the endothelial inflammatory response.

## Materials and Methods

### Reagents

Dulbecco’s phosphate buffered saline (PBS), M199 medium with phenol red, porcine gelatin, 4-hydroxytamoxifen (4-HT) and cyclodextrin-encapsulated 17-β-estradiol (E2) were all from Sigma Aldrich (St Louis, MO). The selective GPR30 agonist G-1 and the specific GPR30 inhibitor G-15 were both from Calbiochem/ EMD Millipore (Darmstadt, Germany). The inhibitor of classical estrogen receptors (ERs) ICI182780, the selective ERα agonist propylpyrazole triol (PPT) and the selective ERβ agonist diarylpropionitrile (DPN) were purchased from Tocris Bioscience (Ellisville, MO). Both control and GPR30 specific siRNAs were from Qiagen (Germantown, MD). M199 medium without phenol red and fetal bovine serum (FBS) were obtained from Gibco/ Invitrogen (Carlsbad, CA). Type 1 Collagenase was purchased from Worthington Biochemical Corporation (Lakewood, NJ). Triton-X-100 and endothelial cell growth supplement (ECGS) were both from VWR International (West Chester, PA).

### Endothelial Cell Isolation and Culture

Second passage human umbilical vein endothelial cells (HUVECs), a widely used model for studying the vascular endothelium, were isolated from human umbilical cords obtained from the Royal Alexandra Hospital in Edmonton, AB. Appropriate procedures for ethics approval and consent were followed as detailed below under ‘Ethics Statement’. Briefly, the umbilical vein was flushed with PBS to remove blood clots and then the HUVECs were isolated using type 1 collagenase dissolved in PBS. The cells were grown in a humidified atmosphere at 37°C with 5% CO_2_/ 95% air in M199 medium with phenol red supplemented by 20% fetal bovine serum (FBS) as well as L-Glutamine (Gibco/ Invitrogen), Penicillin-Streptomycin (Life Technologies) and 1% ECGS. Cells from both male and female infants were used without regard to sex differences. We have previously confirmed the endothelial nature of these cells by immunostaining for the endothelium-specific marker, von Willebrand’s factor (vWF) [22]. Our laboratory has published extensively using HUVECs as a model system for the vascular endothelium [23]–[27].

Once the HUVECs were 80–90% confluent, these were incubated overnight in phenol red free M199 medium (to prevent estrogenic actions of phenol red) prior to use in experiments. A lower degree of confluence was used only for the siRNA experiments.

Cells from a different cord were always used for replicating individual experiments. For example, N = 4 independent experiments indicate that the study was repeated 4 times and cells were used from a different cord in each case.

### Western Blotting

Western blotting was performed on HUVEC lysates. Bands for GPR30 (rabbit polyclonal antibody from Novus Biologicals, Littleton, CO, #NBP1-31239), IκBα (rabbit polyclonal antibody from Cell Signaling, Beverly, MA, #9242) , VCAM-1 (rabbit polyclonal antibody from Santa Cruz Biotechnologies, Santa Cruz, CA, #sc-8304) and ICAM-1 (mouse monoclonal antibody from Santa Cruz Biotechnologies, #sc-8439) were normalized to α-tubulin (rabbit polyclonal antibody from Abcam, Cambridge, MA, #ab15246). Anti-α-tubulin was used at 0.4 µg/ml, while all other antibodies were used at 1 µg/ml. Goat anti-rabbit and Donkey anti-mouse conjugated secondary antibodies were purchased from Licor. The protein bands were detected by a Li-cor Odyssey BioImager and quantified by densitometry using corresponding software Odyssey V3.0 (Li-cor Biosciences, Lincoln, NB). Samples generated from one particular umbilical cord were always run on the same gel. Cell lysates from untreated cells were loaded on each gel and all data were expressed as fold increase over the corresponding untreated control.

### Immunofluorescence

**Figure 1:**
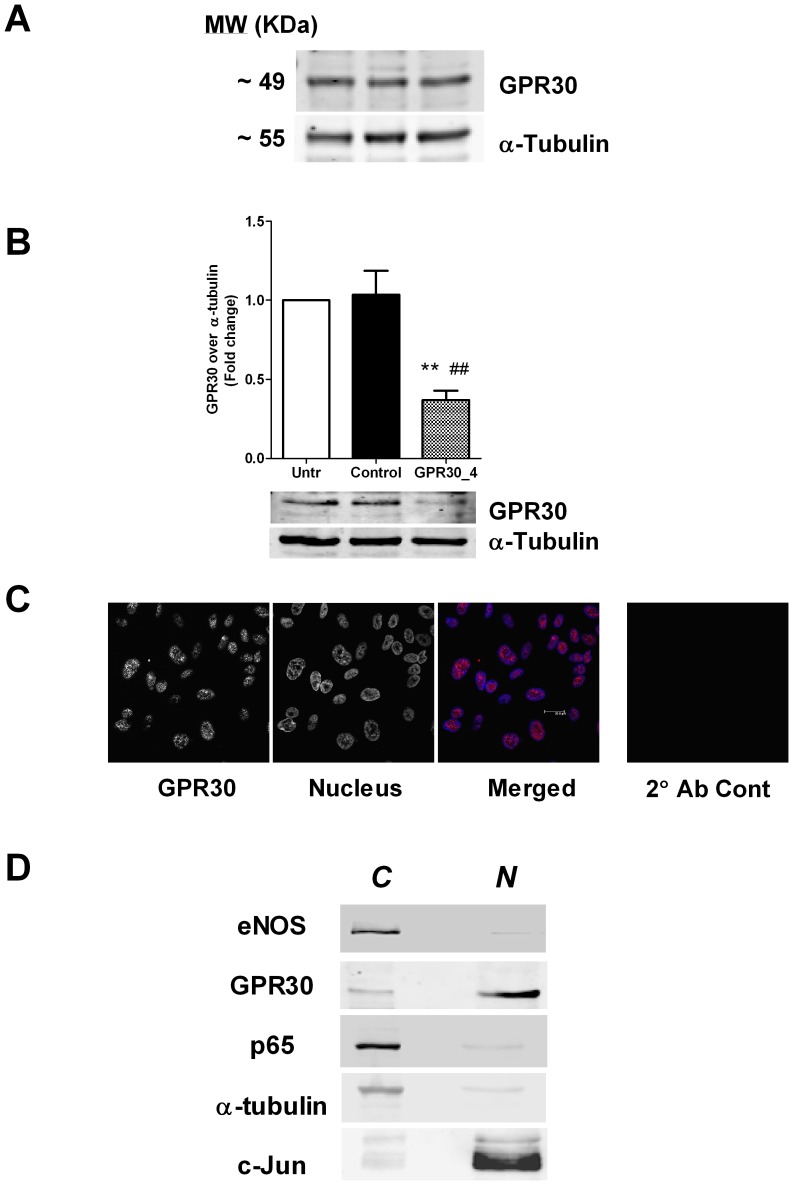
Human endothelial cells express GPR30 protein in the cell nucleus. (A) Confluent monolayers of HUVECs from 3 different cords were lysed and the protein lysates were immunoblotted for GPR30. α-tubulin was used as loading control. (B) HUVEC monolayers at 30–40% confluence were treated with 40 nM siRNA (control or GPR30_4) for 48 hours prior to lysis followed by immunoblotting of the cell lysates for GPR30. α-tubulin was used as loading control. Data shown are mean ± SEM of 4 independent experiments. ** and ## indicate p<0.01 compared to untreated and control siRNA-treated cells, respectively. (C) Confluent HUVECs grown on glass coverslips were fixed, permeabilized and immunostained with anti-GPR30 antibody. Nuclei were stained with Hoechst33342 dye. The merged image shows GPR30 (red) and nuclei (blue) in pseudocolor. Representative images from 3 independent experiments are shown. Bar, 20 µm. (D) Confluent HUVECs were lysed and fractionated into cytosolic (*C*) and nuclear (*N*) fractions prior to western blotting for eNOS, GPR30, p65, α-tubulin and c-Jun. A representative set of images (obtained from different membranes) from 3 independent experiments is shown.

Confocal microscopy was used for immunostaining of GPR30 (Figure 1). Briefly, HUVECs were grown on sodium bicarbonate treated glass coverslips (1.5), fixed in 4% formalin, permeabilized with 0.1% Triton-X-100 in PBS and immunostained with anti-GPR30 antibody (1∶150) similar to our previous publication [22]. The cells were imaged using a Leica SP5 laser scanning confocal microscope (Leica Microsystems Inc, Deerfield, IL) with a 60X water-immersion objective (i.e. 600 times magnification). Data were analyzed with the Leica Application Suite (LAS) software program (Leica Microsystems Inc). Cells treated with a secondary antibody alone were used as a control (as shown in Figure 1C).

For epifluorescence studies (p65 visualization), HUVECs were fixed in 4% formalin, permeabilized with 0.1% Triton-X-100 in PBS and immunostained using overnight incubation with a rabbit polyclonal antibody against p65 (Santa Cruz Biotechnologies, #sc-372; 1∶200). Cells were treated with Alexa Fluor488 (green) conjugated goat anti-rabbit secondary antibody (Molecular Probes, Eugene, OR) for 30 minutes in the dark. Nuclei were attained with the Hoechst33342 nuclear dye also from Molecular Probes. Cells were visualized under an Olympus IX81 fluorescent microscope (Carson Scientific Imaging Group; Markham, Ontario, Canada) using Slidebook 2D, 3D Timelapse Imaging Software (Intelligent Imaging Innovations Inc.; CO). All images presented are in 100 times magnification.

### siRNA Treatment

HUVEC monolayers were used at 30–40% confluence for these experiments. We used control siRNA and a GPR30 specific siRNA (GPR30_4) both at 40 nM concentrations. The siRNAs were initially incubated with Oligofectamine in serum free Optimem1 media for 30 minutes to allow for the formation of siRNA-Oligofectamine complexes. Then the complete media was removed from the HUVECs and the serum free media containing siRNA-Oligofectamine complexes was added in a drop-wise manner. After 6 hours of incubation, half the volume of complete media (M199 with 20% FBS) was added without removing the Optimem1 media. On the next day, the Optimem1 media was removed and replaced with M199 containing 20% FBS. After another 24 hours, the cells were harvested for western blotting studies. The efficacy of knockdown was determined by western blotting as shown in Figure 1B.

### Subcellular Fractionation

Confluent HUVECs were trypsinized and centrifuged to obtain a cell pellet. The pellet was then lysed and subcellular fractionation into nuclear and cytosolic components performed using the NE-PER Nuclear and Cytoplasmic Extraction Reagents kit (Thermo Scientific, Rockford, IL) according to the manufacturer’s instructions. Different proteins present in nuclear and cytosolic compartments were identified by western blotting using specific antibodies such as those against endothelial nitric oxide synthase (eNOS, mouse monoclonal antibody from BD Biosciences, #610297), p65 (rabbit polyclonal antibody from Santa Cruz Biotechnologies, #sc-372) and c-Jun (mouse monoclonal antibody from Millipore, #05- 1076). The purity of nuclear preparation was ascertained by the presence of the nuclear marker c-Jun and the absence of α-tubulin and p65 (as these are unstimulated cells, p65 remains in the cytoplasm). A molecule normally found in cell membrane and the cytoplasm, eNOS, was also detected in the cytosolic but not in the nuclear fraction.

### Ethics Statement

The protocol for human umbilical cord collection and HUVEC isolation was approved by the University of Alberta Ethics Committee and the investigation also conformed to the principles outlined in the Declaration of Helsinki and also Title 45, US Code of Federal Regulations, Part 46, Protection of Human Subjects, effective December 13, 2001. All subjects provided informed consent in writing before inclusion in the study.

### Statistics

All data are presented as the mean ± SEM of between 4 and 8 independent experiments. Each independent experiment was performed using HUVECs from a different umbilical cord. All data are expressed as fold change over the untreated control. One way analysis of variance (ANOVA) was used for determination of statistical significance, with the appropriate post-test (Dunnett’s test for comparison to control, Tukey’s test for multiple comparisons). Repeated measures test was used wherever applicable. The PRISM 5 statistical software (Graph Pad Software, San Diego, CA) was used for all analyses. P< 0.05 was taken as significant.

## Results

### GPR30 Protein is Expressed in Human Endothelial Cells

While several groups have demonstrated the presence of GPR30 protein in the vascular endothelium of rodents [13], [14], its existence in human endothelia has been less clear. To address this question, we first examined for GPR30 expression by immunoblotting HUVEC lysates obtained from 3 different umbilical cords (both male and female). We found a band corresponding to GPR30 with a molecular weight of ∼49 KDa in all the samples (Figure 1A); indicating GPR30 was indeed present in these cells. To further ascertain the identity of this band, we used a GPR30 specific siRNA to knock down the expression of this protein. As shown in Figure 1B, the GPR30 specific siRNA (GPR30_4) but not the non-specific control, significantly reduced GPR30 protein levels in these endothelial cells.

To determine the subcellular localization of GPR30 within the endothelial cells, we performed immunofluorescence of fixed and permeabilized cells. Interestingly, GPR30 staining was confined to the endothelial cell nucleus (Figure 1C). To examine the apparent nuclear location of GPR30 in greater detail, we performed subcellular fractionation of these cells into cytosolic and nuclear components. The bulk of the GPR30 protein was localized into the nuclear fraction (Figure 1D); further confirming that GPR30 was predominantly in the nucleus of these cells. While G-protein coupled receptors have been traditionally shown to be on cell surfaces or associated with the endoplasmic reticulum, several groups have now demonstrated nuclear GPR30 expression in a number of human cell types such as macrophages, regulatory T lymphocytes and breast cancer associated fibroblasts [17], [28]. Our data suggest a potential role for GPR30 in regulating endothelial protein expression, such as under pro-inflammatory conditions.

### Endothelial GPR30 Activation Attenuates Cytokine Induced Inflammatory Response

**Figure 2:**
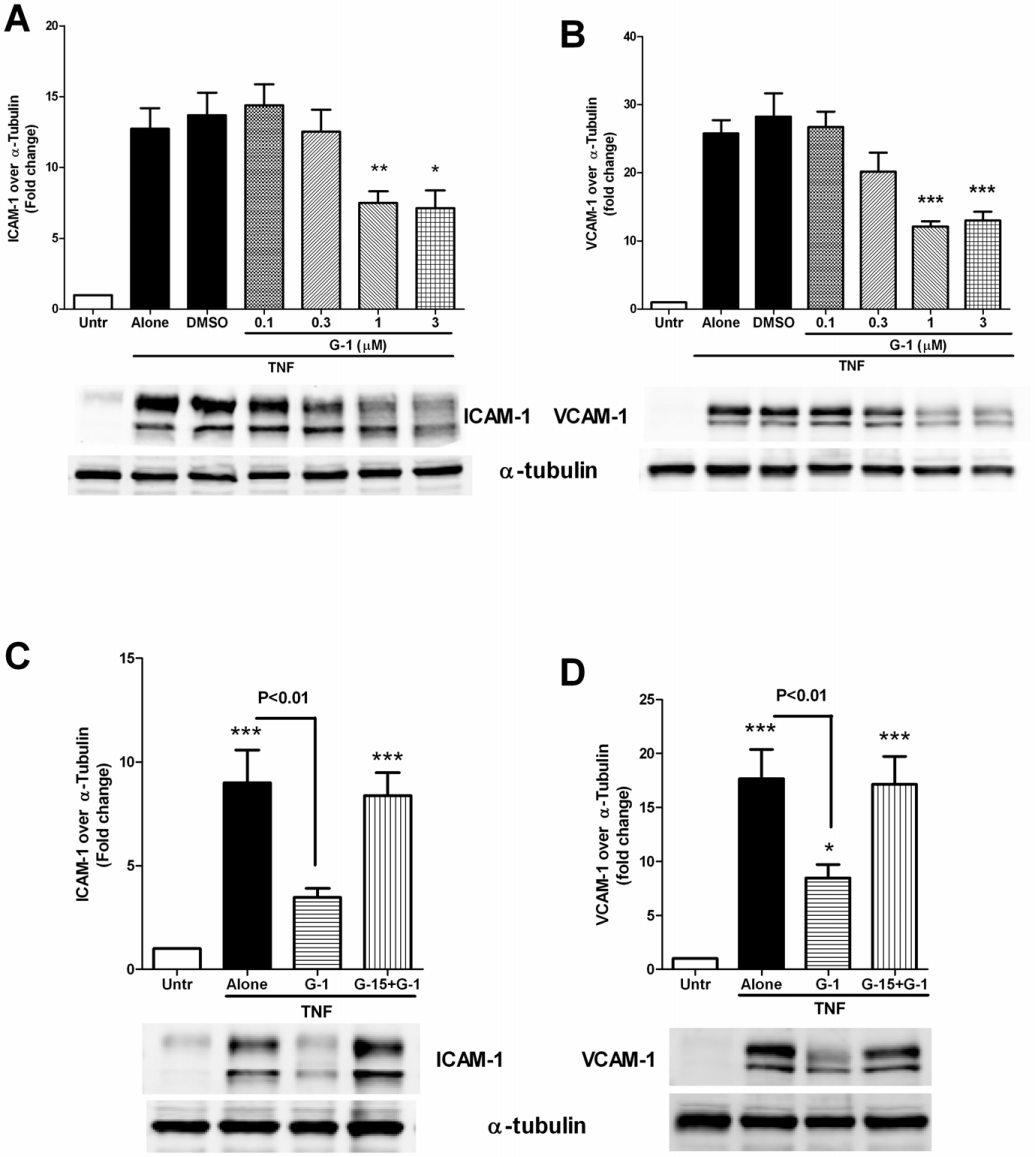
GPR30 activation attenuates TNF-mediated endothelial expression of ICAM-1 and VCAM-1. (A and B) Confluent HUVEC monolayers were pre-treated with the selective GPR30 agonist G-1 (0.1–3 µM) for 45 minutes prior to 4 hour stimulation with TNF (5 ng/ml). Cells were lysed and the lysates were immunoblotted with antibodies against ICAM-1 (A), VCAM-1 (B) and α-tubulin. DMSO (1∶5000) was used as a solvent control for G-1. (C and D) Confluent HUVECs were pre-treated for 30 minutes with/without the specific GPR30 inhibitor G-15 (2 µM) followed by treatment with G-1 (1 µM) for 45 minutes prior to 4 hour stimulation with TNF (5 ng/ml). Cells were lysed and the lysates were immunoblotted with antibodies against ICAM-1 (C), VCAM-1 (D) and α-tubulin. Data shown are mean ± SEM of 6–8 independent experiments. *, ** and *** indicate p<0.05, p<0.01 and p<0.001 respectively, compared to the untreated control.

Tumor necrosis factor (TNF, also called TNF-α), is a major pro-inflammatory cytokine involved in the pathogenesis of atherosclerosis and various other inflammatory diseases [29]. Endothelial cells are activated on exposure to exogenous TNF, causing upregulation of leukocyte adhesion molecules such as ICAM-1 (intercellular cell adhesion molecule-1) and VCAM-1 (vascular cell adhesion molecule-1) which promote leukocyte recruitment and contribute to the progression of the inflammatory process [30]–[32]. Given the importance of adhesion molecules on the pathogenesis of inflammatory conditions, we examined the role of GPR30 activation on TNF-mediated upregulation of these proteins. GPR30 was activated by its selective agonist G-1 while it could be blocked by the selective antagonist G-15. We found that prior activation of GPR30 by G-1 concentration-dependently inhibited TNF-mediated upregulation of both ICAM-1 and VCAM-1 (Figures 2A and 2B). While G-1 at 0.3 µM concentration showed only modest effects, maximal response was obtained at 1 µM G-1, which caused ∼40–60% decrease in levels of these inflammatory molecules. Based on these results, we used G-1 at a concentration of 1 µM for all subsequent studies. To demonstrate that the G-1 effects were actually mediated through GPR30, we blocked the endothelial GPR30 with its selective antagonist G-15 (2 µM), which completely abolished the anti-inflammatory G-1 effects (Figures 2C and 2D). G-15 alone had no effect on TNF induced ICAM-1 and VCAM-1 expression (For ICAM-1, TNF alone: 8.42±1.53; G-15+TNF: 7.90±1.51. For VCAM-1, TNF alone: 15.80±1.25; G-15+TNF: 15.63±1.51. All values are fold change over untreated control; N = 5). These data suggest that G-1 exerts anti-inflammatory effects on TNF stimulated endothelial cells through selective activation of GPR30.

**Figure 3:**
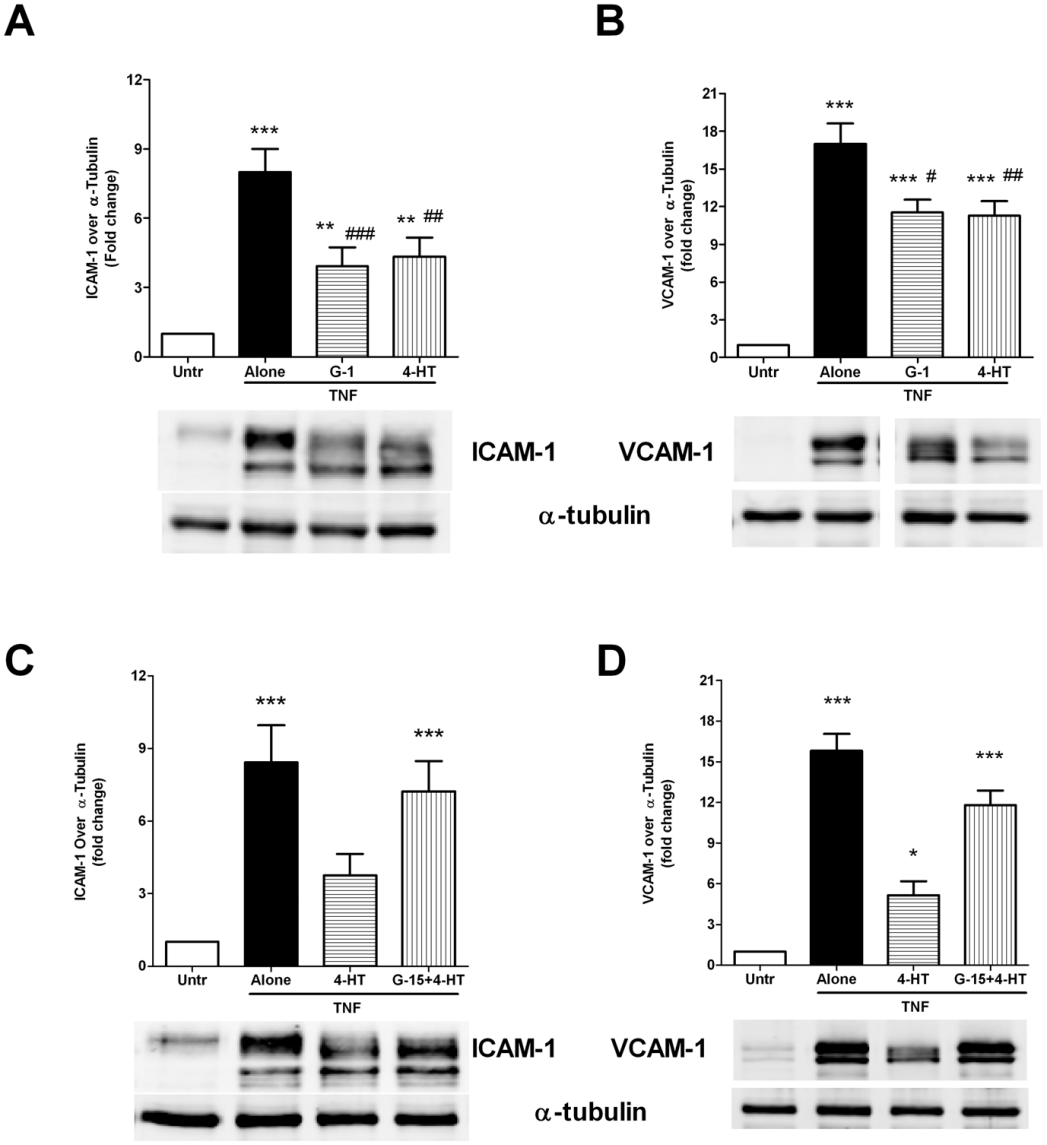
4-hydroxy tamoxifen (4-HT) exerts anti-inflammatory effects similar to G-1. (A and B) Confluent HUVEC monolayers were pre-treated with either G-1 (1 µM) or 4-HT (10 µM) for 45 minutes prior to 4 hour stimulation with TNF (5 ng/ml). Cells were lysed and the lysates were immunoblotted with antibodies against ICAM-1 (A), VCAM-1 (B) and α-tubulin. Data are shown as mean ± SEM of 4 independent experiments. (C and D) Confluent HUVECs were pre-treated with G-15 (2 µM) followed by treatment with 4-HT (10 µM) for 45 minutes prior to 4 hour stimulation with TNF (5 ng/ml). Cells were lysed and the lysates were immunoblotted with antibodies against ICAM-1 (C), VCAM-1 (D) and α-tubulin. Data are shown as mean ± SEM of 5 independent experiments. ** and *** indicate p<0.01 and p<0.001 respectively, compared to the untreated control. #, ## and ### indicate p<0.05, p<0.01 and p<0.001 respectively, compared to TNF alone.

To further examine the role of GPR30, we used 4-hydroxytamoxifen (4-HT), an ERα inhibitor with GPR30 activating properties in similar studies. Due to the lack of exogenous estradiol (E2) in our system, 4-HT was expected to function only as a GPR30 agonist under these conditions. Indeed, pre-treatment with 4-HT (10 µM) partially attenuated TNF mediated ICAM-1 and VCAM-1 expression (Figures 3A and 3B) to a similar extent as the selective agonist G-1. These effects of 4-HT were also blocked by GPR30 inhibitor G-15, suggesting a specific role for GPR30 (Figures 3C and 3D).

### Cytokine Induced NF-κB Activation is Unaffected by GPR30

**Figure 4:**
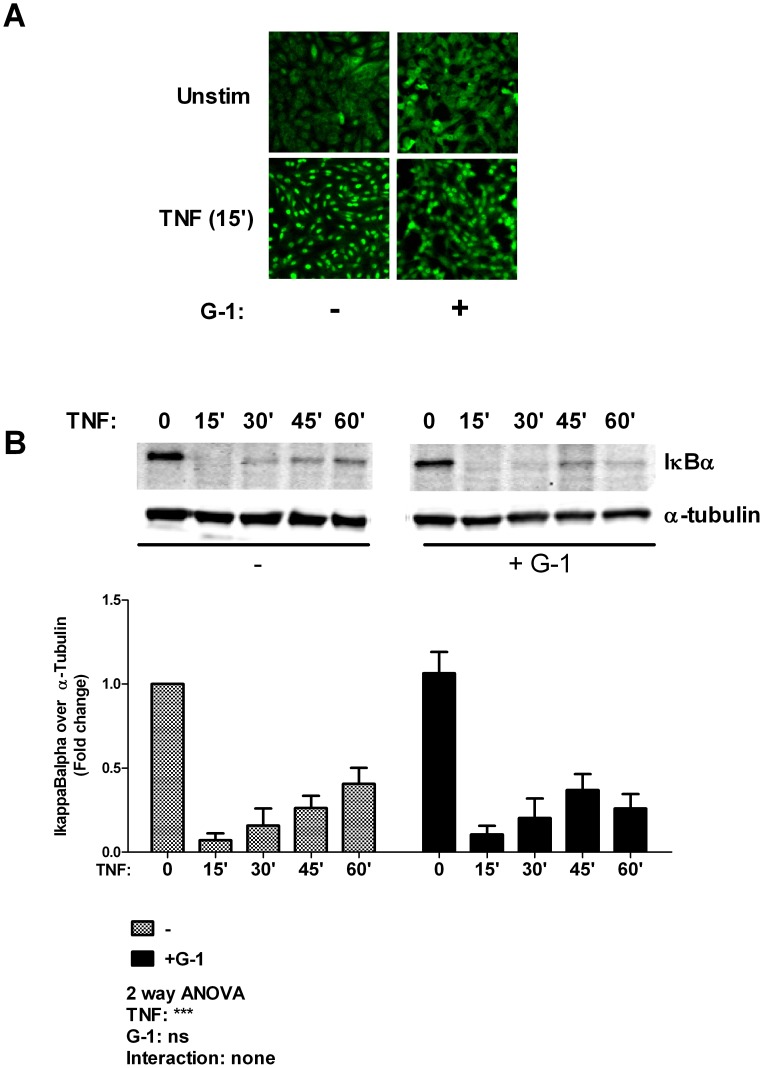
G-1 does not alter TNF-mediated endothelial activation of NF-κB. (A) Confluent HUVEC monolayers were pre-treated with/without G-1 (1 µM) for 45 minutes prior to 15 minute stimulation with TNF (5 ng/ml). Cells were immediately fixed, permeabilized and then immunostained with anti-p65 antibody. Representative images from 3 independent experiments are shown. Magnification (X100). (B) Confluent HUVECs were pre-treated with/without G-1 (1 µM) for 45 minutes prior to stimulation with TNF (5 ng/ml) for the indicated time periods. Cells were lysed and the lysates were immunoblotted with antibodies against IκBα and the loading control α-tubulin. Data are summarized as mean ± SEM of 3 independent experiments. *** indicates p<0.001.

NF-κB is a major transcriptional pathway that regulates the expression of many pro-inflammatory molecules including TNF-induced upregulation of both ICAM-1 and VCAM-1 [33], [34]. The activation of NF-κB was determined by the degradation of IκBα and the nuclear translocation of p65. We found that TNF stimulation promoted rapid degradation of IκBα and nuclear translocation of p65, neither of which was altered by prior treatment with the GPR30 agonist, G-1 (Figures 4A and 4B). This lack of effects (on NF-κB activity) suggests that the GPR30 effects on the endothelial inflammatory response are likely to be independent of NF-κB activation.

### Estradiol Lacks the Anti-inflammatory Properties of GPR30 Agonists

**Figure 5:**
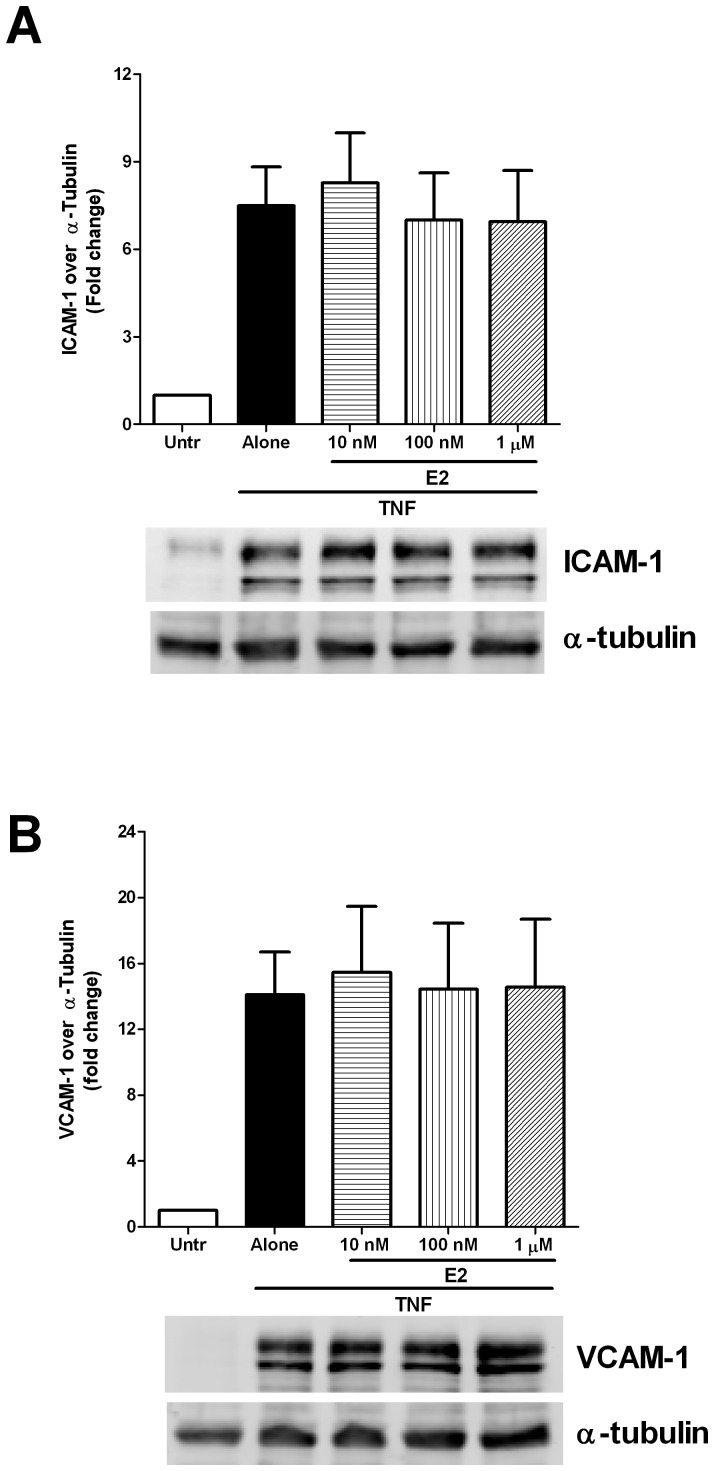
17-β-estradiol (E2) alone has no effect on TNF-mediated endothelial ICAM-1 and VCAM-1 expression. Confluent HUVEC monolayers were pre-treated with different concentrations (10–1000 nM) of E2 for 45 minutes prior to 4 hour stimulation with TNF (5 ng/ml). Cells were lysed and the lysates were immunoblotted with antibodies against ICAM-1 (A), VCAM-1 (B) and α-tubulin. Data are shown as mean ± SEM of 4 independent experiments.

Since E2 acts through 3 different receptors, ERα, ERβ as well as GPR30 (all 3 of which are expressed in HUVECs); we next examined if E2 alone could mimic the effects of GPR30 activators. Surprisingly, E2 at a wide range of concentrations (10 nM to 1 µM) had no effect at all on TNF-mediated upregulation of ICAM-1 and VCAM-1 in the HUVECs (Figures 5A and 5B). Since similar concentrations of E2 have been shown to activate GPR30 in cultured cells, these data suggest that either E2 was unable to activate GPR30 in HUVECs or that signaling through the classical ERs prevented the GPR30 effects.

### Classical Estrogen Receptors Appear to Negate the Anti-inflammatory Effects of GPR30

**Figure 6:**
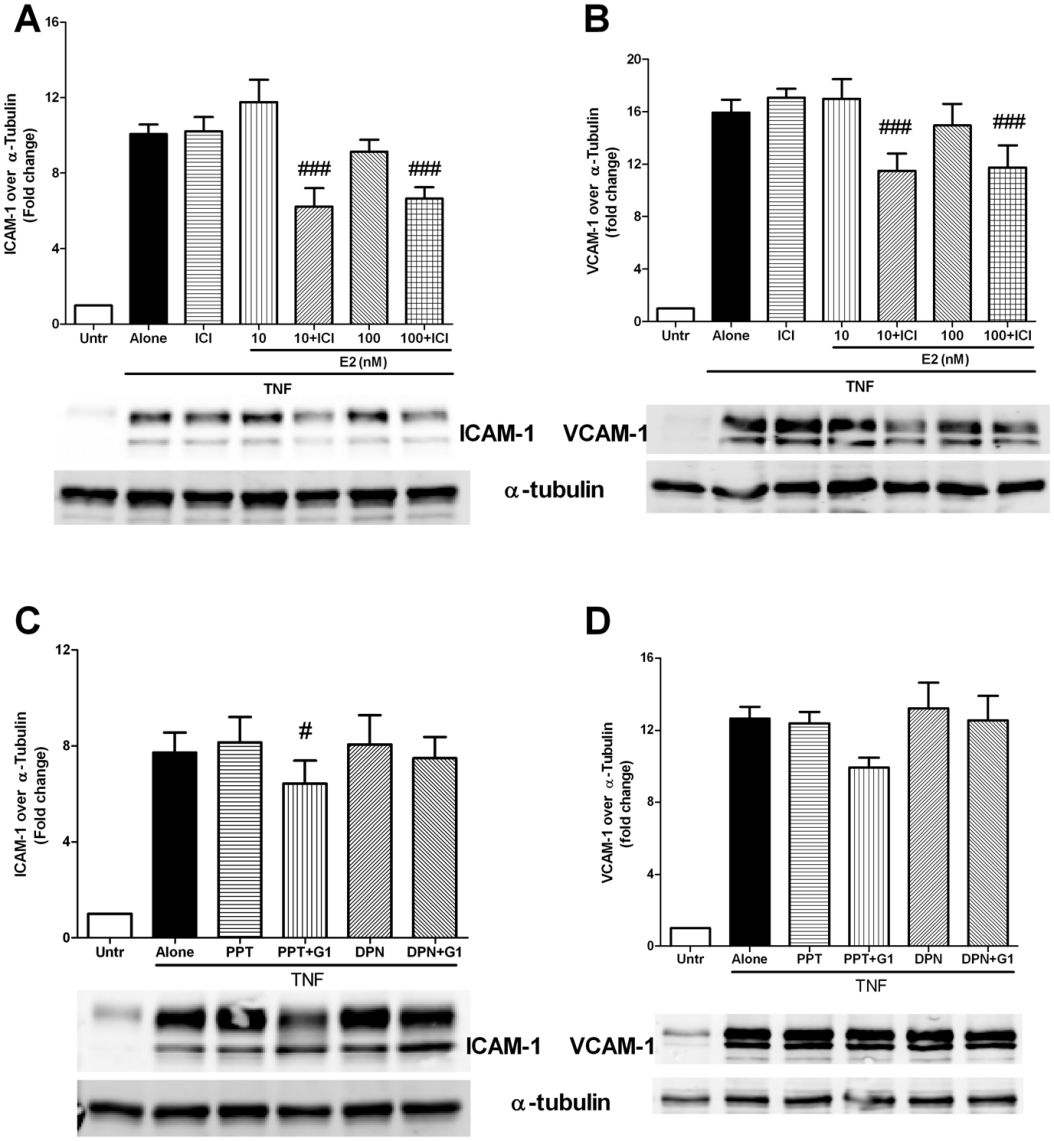
Classical estrogen receptors (ERs) antagonize GPR30 mediated anti-inflammatory effects on the TNF-treated endothelium. (A and B) Confluent HUVECs were pre-treated with/without the classical ER blocker ICI 182780 (ICI, 1 µM) for 30 minutes prior to 45 minute treatment with E2 (10–100 nM). The cells were then stimulated with TNF (5 ng/ml) for 4 hours. Cell lysates were prepared and immunoblotted with antibodies against ICAM-1 (A), VCAM-1 (B) and α-tubulin. (C and D) Confluent HUVECs were pre-treated with/without the ERα agonist PPT (10 nM) or ERβ agonist DPN (10 nM) for 30 minutes followed by 45 minutes with/without the GPR30 agonist G-1 (1 µM). Cells were then stimulated with TNF (5 ng/ml) for 4 hours, lysed and immunoblotted with antibodies against ICAM-1 (C), VCAM-1 (D) and α-tubulin. Data are shown as mean ± SEM of 6–7 independent experiments. # and ### indicate p<0.05 and p<0.001 respectively, compared to TNF alone.

We then examined the role for different ERs (classical and GPR30) in mediating anti-inflammatory effects. To determine if exogenous E2 could actually activate GPR30, we pre-treated HUVECs with the classical ER antagonist ICI182780 prior to E2 treatment and TNF stimulation. In presence of ICI182780, E2 effects, if any, would be mediated through its non-classical receptor GPR30 rather than through ERα or ERβ. We found that E2 in the presence of ICI182780 significantly reduced the TNF induced ICAM-1 and VCAM-1 expression (Figures 6A and 6B), suggesting an unmasking of the anti-inflammatory GPR30 signaling under these conditions.

Although ICI182780 has been described as a potential agonist for GPR30 [35], we did not observe any inhibitory effects of this compound on TNF stimulated ICAM-1 and VCAM-1(Figures 6A and 6B). Similar lack of effects were also observed over a range of concentrations (0.1–10 µM) of ICI182780 in presence of TNF (data not shown). Our findings are in accordance with results from the study by Balhuizen et al where ICI182780 failed to elicit GPR30 dependent effects in contrast to equimolar concentrations of G-1 [36]. The differences in chemical structure between the two compounds and availability in intracellular compartments might account for this discrepancy.

Finally, we examined the interactions between signaling through classical ERs and GPR30 using selective receptor agonists. We used PPT for ERα and DPN for ERβ, either alone or in combination with the GPR30 agonist G-1 prior to TNF stimulation of endothelial cells. Interestingly, G-1 still had a minor inhibitory effect (∼10–15%) on ICAM-1 (but no significant effects on VCAM-1) expression in presence of PPT; while the presence of DPN completely abolished the G-1 effects on both ICAM-1 and VCAM-1 upregulation (Figures 6C and 6D). The minor effect of G-1 in presence of PPT is quite different from that of G-1 alone and would presumably have little biological significance on inflammation. Neither PPT nor DPN, when used alone, had any effects on the expression of adhesion molecules. These findings indicate a novel role for classical ERs in antagonizing the GPR30 dependent anti-inflammatory effects in the endothelium.

## Discussion

In this paper, GPR30 protein expression was demonstrated in cultured human endothelial cells with predominant localization to the cell nuclei. Activation of endothelial GPR30 attenuated TNF induced upregulation of the pro-inflammatory leukocyte adhesion molecules, suggesting an anti-inflammatory role for this receptor. Interestingly, estrogen alone did not elicit similar anti-inflammatory effects, likely due to opposing actions of the classical ERs and GPR30 on the inflammatory process.

GPR30 is present in the vascular endothelium of both mice and rats where it appears to exert potentially beneficial effects [13], [14]. While the GPR30 mRNA has been shown in human endothelial cells and responses to GPR30 agonist have been documented [19], [20], the actual GPR30 protein has not been demonstrated in most publications. We have now clearly shown the presence of GPR30 by immunoblotting and immunofluorescence in cultured human endothelial cells. This endothelial GPR30 also appears to be largely confined to the nucleus. Our findings of nuclear GPR30 are in line with similar findings in other human cell types such as T lymphocytes, fibroblasts and macrophages [17], [28]. On the other hand, a recent paper by Li et al has shown the presence of GPR30 protein in the cytoplasm of rat aortic endothelial cells [37]. However, a similar species-based difference in subcellular localization of GPR30 has been previously described. Blasko et al have shown that while GPR30 is intranuclear in human macrophages it is mostly cytosolic in the murine RAW macrophage cell line, which is analogous to our findings [17]. The presence of GPR30 in the endothelial nucleus suggests a potential regulatory role on cellular functions such as cell division and protein upregulation during inflammatory stimulation.

TNF is a major pro-inflammatory cytokine involved in the pathogenesis of atherosclerosis and many other inflammatory diseases [29], [38], [39]. TNF induces upregulation of leukocyte adhesion molecules such as ICAM-1 and VCAM-1 on the endothelium, involving activation of the NF-κB pathway [33]. Both ICAM-1 and VCAM-1 are expressed at low levels on the resting endothelium. Increased expression of these adhesion molecules causes enhanced interactions with leukocytes which are subsequently recruited in a stepwise manner involving rolling, activation, firm adhesion and transmigration from the bloodstream into extravascular tissues, further contributing to inflammation [40]–[42]. While the inflammatory response is indeed necessary for the body’s defenses against invading micro-organisms and to promote wound healing, excessive and/or uncontrolled inflammation is usually detrimental, contributing to inflammatory diseases such as atherosclerosis. Targeting the upregulation of ICAM-1 and VCAM-1 can attenuate the inflammatory effects; hence, leukocyte-endothelial interactions have been the subjects of intense study for developing novel anti-inflammatory therapies. Our work suggests a novel role for GPR30 in attenuating the TNF-induced upregulation of ICAM-1 and VCAM-1. We found that GPR30 activation, even at the highest concentrations of the agonist used could only have a partial (∼40–60%) effect on these molecules, which could be potentially beneficial in controlling excessive inflammation without compromising the body’s defensive mechanisms. The effects mediated by the selective GPR30 agonist were also completely blocked by the GPR30 inhibitor G-15, suggesting the involvement of a specific GPR30 dependent mechanism. Interestingly, no effect of GPR30 was observed on TNF mediated activation of the transcription factor NF-κB. Previous studies from several groups have demonstrated a critical role for this pathway in upregulating both ICAM-1 and VCAM-1 in TNF stimulated endothelium [33], [34]. A recently published study from our own group also suggests an essential role for NF-κB on these pathways [43]. Since NF-κB is indeed involved in TNF mediated expression of leukocyte adhesion molecules, our results suggest that the GPR30 dependent effects are likely exerted downstream of NF-κB activation. GPR30 effects might be exerted at the levels of transcription factor binding, mRNA stability or protein transcription, all of which are downstream of NF-κB activation and could be interesting topics of future research in this area. Indeed, work by Holm et al has already indicated the ability of GPR30 to prevent endothelial DNA synthesis which may contribute to its effects on pro-inflammatory protein expression [20].

The role of estrogen on the inflammatory process has been quite controversial despite many studies involving human subjects, human cells/tissues and rodent models of disease. An anti-inflammatory role for E2 was originally suggested based on the relative cardiovascular protection of premenopausal women compared to age-matched men [1], [2]. Studies using exogenous E2 in animal models have also suggested beneficial effects against pro-inflammatory and atherosclerotic changes [44]–[47]. Cell culture models involving human cells have given more equivocal results with both anti-inflammatory and pro-inflammatory roles suggested for E2 [48]–[52]. In contrast, results from clinical trials of estrogen-based hormone replacement therapies have been quite disappointing in mitigating cardiovascular disease. It is now increasingly apparent that estrogen signaling in the vascular system is a complex phenomenon with different receptors and signaling pathways, which can be altered depending on cell type, concentration of estradiol and other confounding factors such as aging, oxidative stress and blood glucose levels [53]–[58]. Indeed, we found that E2 alone did not elicit anti-inflammatory effects similar to the GPR30 agonist in our cultured endothelial cells. Previous findings from our group have shown that the HUVECs also express the classical ERs ERα and ERβ both of which appear to exert downstream effects such as eNOS upregulation [27], [59]. Given the presence of multiple functional ERs, we next examined the interactions among these different receptors. Interestingly, blockade of the classical ERs by ICI182780 in the presence of exogenous E2 significantly decreased the TNF induced inflammatory protein expression, suggesting that signaling through classical ERs might be involved in masking the beneficial GPR30 effects. Similarly, concomitant activation of GPR30 and either ERα or ERβ could prevent the attenuation of endothelial inflammation, demonstrating a direct inhibitory effect of classical ERs on GPR30 mediated activity. While little is known about the complex interactions among the 3 ERs in different cell systems, work by Ding et al has demonstrated opposing actions of ERα and GPR30 on ERK phosphorylation in cultured vascular smooth muscle cells (VSMCs). Cultured VSMCs with low levels of GPR30 expression showed inhibition of ERK phosphorylation in presence of E2, presumably mediated through the classical ERs. Transfection with GPR30, however, reversed these effects with E2-induced ERK phosphorylation, suggesting contradictory effects of GPR30 and classical ERs on the ERK pathway [60]. Another study by Gao et al has recently shown opposing effects of GPR30 and classical E2 signaling on cell proliferation in mouse uterine tissue involving modulation of ERK phosphorylation [61]. In a similar vein, our results indicate opposing effects of GPR30 and the classical ERs in regulating the endothelial inflammatory response. Relative expression of the 3 ERs may determine the precise biological effects of exogenous E2 administration in such a system, with beneficial anti-inflammatory effects associated with the increased presence of functional GPR30.

The potential limitations of our study include the use of a single cell type, namely, HUVECs and a single pro-inflammatory cytokine, TNF. Given the differences in endothelial cells from different vascular beds, future work needs to examine the roles of GPR30 in other endothelial cell types to better understand its roles in inflammatory conditions affecting different tissues and organs. Similarly, the antiinflammatory effects observed on TNF stimulation need to be validated in the context of other pro-inflammatory stimuli, namely, cytokines, chemokines and/or oxidized low density lipoprotein (oxLDL).

Based on the potential anti-inflammatory role of GPR30, further studies of this novel estrogen receptor are needed to understand the complexity of vascular estrogen signaling. Future research in this area can lead to the development of receptor-specific targeted approaches to harness the beneficial estrogen effects while potentially minimizing the harmful side-effects.
